# Die hard: timberline conifers survive annual winter embolism

**DOI:** 10.1111/nph.16304

**Published:** 2019-11-23

**Authors:** Stefan Mayr, Peter Schmid, Barbara Beikircher, Feng Feng, Eric Badel

**Affiliations:** ^1^ Department of Botany University of Innsbruck Sternwartestr. 15 6020 Innsbruck Austria; ^2^ College of Forestry Northwest A&F University 3 Taicheng Rd Yangling 712100 Shaanxi China; ^3^ INRA, PIAF Université Clermont Auvergne F‐63000 Clermont–Ferrand France

**Keywords:** cavitation fatigue, embolism, freeze–thaw, frost drought, micro‐CT, Norway spruce, timberline conifer, xylem refilling

## Abstract

During winter, timberline trees are exposed to drought and frost, factors known to induce embolism. Studies indicated that conifers cope with winter embolism by xylem refilling. We analysed the loss of hydraulic conductivity (LC) in *Picea abies* branch xylem over 10 years, and correlated winter embolism to climate parameters. LC was investigated by direct X‐ray micro‐computer tomography (micro‐CT) observations and potential cavitation fatigue by Cavitron measurements. Trees showed up to 100% winter embolism, whereby LC was highest, when climate variables indicated frost drought and likely freeze–thaw stress further increased LC. During summer, LC never exceeded 16%, due to hydraulic recovery. Micro‐CT revealed homogenous embolism during winter and that recovery was based on xylem refilling. Summer samples exhibited lower LC in outermost compared to older tree rings, although no cavitation fatigue was detected. Long‐term data and micro‐CT observations demonstrate that timberline trees can survive annual cycles of pronounced winter‐embolism followed by xylem refilling. Only a small portion of the xylem conductivity cannot be restored during the first year, while remaining conduits are refilled without fatigue during consecutive years. We identify important research topics to better understand the complex induction and repair of embolism at the timberline and its relevance to general plant hydraulics.

## Introduction

Xylem embolism is a major risk for plants as it impairs the water supply of distal tissues (Tyree & Zimmermann, 2002; McDowell *et al.*, 2008). Plants cope with this risk by respective hydraulic safety and efficiency of the xylem (Choat *et al.*, 2012; Gleason *et al.*, 2016). Embolism on summer drought thus is rare but potentially lethal because repair (i.e. xylem refilling; Zwieniecki & Holbrook, 2009; Brodersen *et al.*, 2010; Earles *et al.*, 2016) usually is impossible (Choat *et al.*, 2015; Charrier *et al.*, 2016; Niu *et al.*, 2017; Lamarque *et al.*, 2018). By contrast, some studies reported winter conditions at high elevation to induce dramatic loss of hydraulic conductivity (LC) due to embolism and suggested xylem refilling to be essential for the survival of timberline trees (Mayr *et al.*, 2003b, 2014).

During winter, timberline trees are exposed to frost drought and freeze–thaw events, both factors known to induce embolism (Sparks & Black, 2000; Mayr *et al.*, 2003b, 2006a). On drought stress, decreasing water potential (Ψ) causes air entry into xylem conduits via the pits when critical limits in Ψ are reached. It was suggested that Ψ inducing more than 50% LC are lethal for conifer species (Brodribb & Cochard, 2009; Brodribb *et al.*, 2010; Choat *et al.*, 2018; but also see Hammond *et al.*, 2019). At the timberline, trees suffer from frost drought, because the ice in soil, roots and frozen trunk parts block any uptake of soil water for months, while cuticular transpiration causes relevant water losses (Mayr *et al.*, 2003b; Duursma *et al.*, 2019). This is especially true in evergreen conifers as needles can reach substantially higher temperatures than the air during sunny winter days (Mayr *et al.*, 2006b). Moreover, the crown is exposed to numerous freeze–thaw cycles, causing embolism even in conifers with small and thus resistant tracheids (Mayr *et al.*, 2003a; Pittermann & Sperry, 2006; Sevanto *et al.*, 2012). Hydraulic dysfunction caused by freeze–thaw events is a common phenomenon in plants growing in seasonal or high elevation environments. Air bubbles formed during the freezing of xylem sap may expand as the sap melts and low Ψ values are re‐established. Experimental as well as field data indicated the combination of frost drought and freeze–thaw stress to be responsible for pronounced winter embolism in conifers at higher elevation (Sparks & Black, 2000; Mayr *et al.*, 2003b, 2006a, 2014).

Here we review a unique 10‐year series of field‐data on embolism formation which demonstrates the surprisingly efficient repair in *Picea abies* growing at the timberline. Results show that this timberline conifer is able to survive annual cycles of extreme Ψ and winter embolism. Correlation with climate data indicate that winter embolism is induced by frost drought, and X‐ray micro‐computer tomography (micro‐CT) analyses prove that spring recovery is based on xylem refilling. Timberline trees are thus an impressive example for survival of embolism based on periodic refilling. In the following, we present and discuss the main results, which may be the base for important future research aims summarized in the conclusion.

## Materials and Methods

### Materials

Measurements for the monitoring of embolism and Ψ and for X‐ray analyses were made on sun‐exposed branches of 2–4 m tall *Picea abies* trees growing at Birgitz Köpfl, Tirol, Austria (2035 m; 47°11′N, 11°19′E). At each sampling date three to five trees were sampled, whereby one, up to 1 m long branch per tree was selected. Hydraulic measurements for monitoring of native embolism and Ψ (three replicate measurements each) were averaged for each branch, which thus is the statistical unit throughout the study. X‐ray micro‐CT analyses were performed on branches previously used for hydraulic measurements, whereby samples adjacent to segments used for hydraulic measurements were prepared. Sampling for cavitation fatigue experiments was done at Praxmar, Tirol Austria (2100 m, 47°80′90″N, 11°80′70″E) during autumn, and branches were fully hydrated for 24 h before use.

### Meteorology

Data from climate stations near the study site at Birgitz Köpfl (Mount Patscherkofel 2252 m provided by ZAMG Zentralanstalt für Meteorologie und Geodynamik, Austria; Lämmerbichlalm 2020 m provided by Lawinenwarndienst Tirol, Austria) were used to calculate the following parameters: mean air temperature and sunshine duration in early winter (November–December) and late winter (January–April), cumulative precipitation (September–December and January–April), cumulative sunshine duration (November–April), number of days with freeze–thaw cycles and permanent snow cover during winter months (September–June). The number of days with freeze–thaw cycles was estimated by counting days with a daily minimum temperature lower than −3°C and daily sunshine duration higher than 50% of maximum day length. The snow cover duration was defined as the number of consecutive days with a minimum snow height of 10 cm.

### Monitoring of embolism and water potential

Measurements of Ψ and LC were conducted from winter 2003/2004 to summer 2013. Sampling was made between 10:00 h and 14:00 h in intervals (3–11 and 1–4 times in winter and summer seasons, respectively). For Ψ determination, distal twigs (*c.* 10 cm) of selected branches (see earlier), were cut, wrapped in plastic bags and measured with a Scholander apparatus in the laboratory (Model 1000; PMS, Albany, OR, USA). For LC measurements, *c.* 4 cm long stem segments (diameter 5–8 mm) were cut from branches and LC quantified with a Xyl'em system (Bronkhorst, Montigny les Cormeilles, France) by measuring the increase in hydraulic conductivity at 4 kPa after removal of xylem embolism by repeated high pressure flushes at 80 kPa (Sperry *et al.*, 1988). Sample preparation followed a previously described protocol (Mayr *et al.*, 2002, 2003b): segments were cut under water and the bark removed at the basal end, before samples were sealed in silicon tubes of the hydraulic system. Flushing and conductivity measurements were carried out with distilled, filtered (0.22 mm), and degassed water containing 0.005% (v/v) Micropur (Katadyn Products Inc., Wallisellen, Switzerland) to prevent microbial growth (Sperry *et al.*, 1988).

### Direct X‐ray micro‐CT visualization of wood tissue embolism

Branch segments (length *c.* 10 cm) were wrapped in parafilm and rapidly sent to INRA facility (PIAF Laboratory, Clermont‐Ferrand, France). There, samples were dropped in liquid paraffin wax in order to prevent drying during the scan and placed in the X‐ray microtomograph (Nanotom 180 XS; GE, Wunstorf, Germany) as described in Cochard *et al.* (2015). The field of view of the micro‐CT scan was adjusted to 8.0 × 8.0 × 8.0 mm^3^ to fit the branches diameter and the X‐ray source set to 60 kV and 240 μA. For each 21‐min scan, 1000 images were recorded during the 360° rotation of the sample. After the three‐dimensional (3D) reconstruction process, the spatial resolution of the final image was 4.00 μm × 4.00 μm × 4.00 μm per voxel. Transverse two‐dimensional (2D) slices of 3D volumes were extracted from the middle of the volume using VGStudio Max© software (Volume Graphics, Heidelberg, Germany) in order to visualize the tissue embolism. For each region of interest, image analysis was performed using imagej software (Schneider *et al.*, 2012) in order to locally measure the level of embolism. For gymnosperms like here, the process consists in a segmentation operation that separates the embolized tracheids from the water‐filled tracheids. Then, we computed the percentage of area with embolized tracheids versus the total area of the region of interest (Choat *et al.*, 2015; Torres‐Ruiz *et al.*, 2016). Overall, three winter samples (one harvested 8 April 2013, two 6 May 2013) and two summer samples (harvested 21 August 2013 and 25 September 2013) were analysed, whereby the embolized vs functional tracheid area was calculated for each tree ring, respectively.

### Cavitation fatigue

Vulnerability curves were measured with the Cavitron technique (Cochard *et al.*, 2005; Beikircher *et al.*, 2010). Branch segments were fixed in a 280 mm rotor in the centrifuge (Sorvall RC‐5; Thermo Fisher Scientific, Waltham, MA, USA) and reservoirs filled with distilled, filtered (0.22 μm) and degassed water containing 0.005% (v/v) Micropur (Katadyn), temperature was set to 10°C. Samples were equilibrated for 20 min at −0.25 MPa, before the rotational speed was increased in 0.5 MPa steps and hydraulic conductance measured. The moving meniscus in the upstream cuvette was observed using a camera (Motic MC 2000; Motic China Group Co. Ltd, Nanjing, Jiangsu, China) and the hydraulic conductance calculated. LC was calculated from the ratio of actual to the maximum hydraulic conductance (Sperry *et al.*, 1988). Curves of each sample were fitted as LC = 100/(1 + exp(*a* (Ѱ – ѰLC50))), where ѰLC50 is *Ψ* at 50% LC (Pammenter & Vander Willigen, 1998). Cavitation fatigue was tested by stopping vulnerability analyses after reaching 50% or 100% LC, rehydrating samples via vacuum infiltration and subsequently re‐measuring a complete vulnerability curve. Branches at 50% LC were vacuum infiltrated in measurement solution for 12 h and branches at 100% LC for 24 h. Preliminary test experiments revealed that these times were necessary to restore maximum conductivity.

### Statistics

Values were first averaged for each branch and the mean ± SE per date calculated. Correlations were tested at 0.05 probability level (SPSS 24, IBM, Ehningen, Germany) with Pearson´s correlation coefficient in case of normal distribution or with Spearman´s correlation coefficient in case of nonnormal distribution.

## Results and Discussion

### Formation of winter embolism

**Figure 1:**
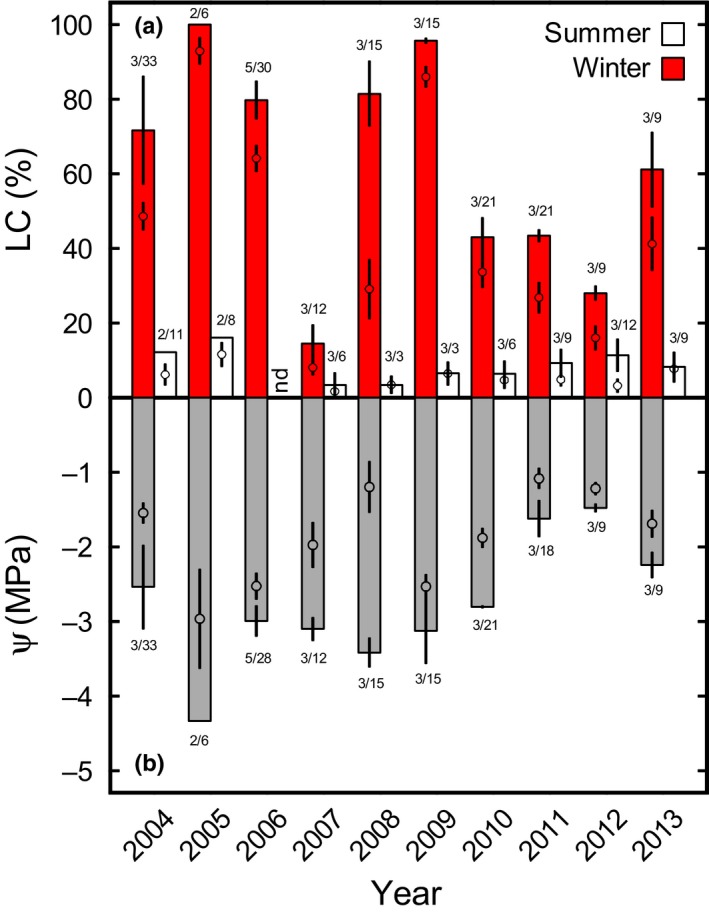
Xylem embolism in winter and subsequent summer seasons. Measurements on medial sections of sun‐exposed branches were taken from January to April and July to October, respectively. (a) Percentage loss of hydraulic conductivity (LC). Maximum LC (i.e. mean ± SE at the sampling date with highest LC) is indicated by bars, dots represent the four months mean. No summer data are available for 2006 (not determined, nd). (b) Water potential (Ψ). Respective minimum (i.e. mean ± SE at the sampling date with lowest Ψ) and mean Ψ are given for winter months. Summer Ψ are not given as influenced by transpiration. Small numbers indicate *n* for maximum/four months values (in few cases only two branches could be measured for maximum LC or minimum Ψ). Parts of 2004, 2005, 2010 and 2011 data were extracted from Mayr *et al.* (2006a,b, 2014). Mean ± SE.

The long‐term data of mean and maximum LC collected between winter 2003/2004 and summer 2013 at the alpine timberline (Fig. 1) demonstrate that winter LC occurs frequently and can be dramatically high, as illustrated by Norway spruce that suffers from high embolism during most winters. Significant embolism with maximum LC > 60% was observed in 6 out of 10  years, whereby maximum and mean LC of each winter season, respectively, correlated (Supporting Information Fig. S1). In the winters of 2005 and 2009, even the 4 months mean (January–April) of LC was as high as 92.9 ± 3.5 and 86.0 ± 2.8%, respectively (Table 1). Only during the winter of 2007, embolism remained negligible, probably due to an overall warm winter with short snow cover duration and an extraordinary warm period in December (Table 1). One might speculate that climate warming will more often lead to winter conditions like in 2007, as average temperatures in the Alps have increased more rapidly than the global trend in recent decades (Böhm *et al.*, 2001; Rebetez & Reinhard, 2007). However, due to the complexity of mountain systems, no general trend in annual precipitation was observed, although critical shifts in seasonal patterns are expected, which may cause more frequent spring droughts (Beniston, 2005; Ciccarelli *et al.*, 2008; Elkin *et al.*, 2013).

**Table 1:** Embolism and climate from winter 2003/2004 to summer 2013.

	2004	2005	2006	2007	2008	2009	2010	2011	2012	2013	*r*
LC_max_ (%)	71.6	100.0	79.8	14.5	81.5	95.7	43.0	43.4	28.0	61.11	
LC_J–A_ (%)	48.6	92.9	64.1	8.1	29.1	86.0	33.7	26.8	16.0	41.2	0.889*
Ψ_min_ (MPa)	−2.5	−4.3	−3.0	−3.1	−3.4	−3.1	−2.8	−1.6	−1.5	−2.2	−0.605
*T* air (°C)											
Nov_‐1_	−0.2	−3.4	−4.0	−0.9	−5.0	−3.2	−0.8	−3.3	2.1	−0.6	−0.640*
Dec_‐1_	−4.8	−3.8	−9.0	−2.1	−5.6	−6.4	−8.1	−9.3	−5.5	−7.5	−0.106
Jan	−9.0	−7.7	−7.2	−3.9	−4.4	−7.2	−8.9	−6.7	−7.3	−6.7	−0.260
Feb	−6.7	−11.6	−8.1	−3.8	−3.6	−9.0	−8.4	−4.7	−11.8	−10.8	−0.244
Mar	−5.4	−4.8	−6.8	−4.1	−6.6	−6.4	−6.1	−3.1	−1.7	−5.8	−0.636*
Apr	−2.0	−1.6	−1.6	3.2	−3.2	0.7	−1.1	1.3	−2.1	0.0	−0.465
Sunshine (h)											
Nov_‐1_	144	90	129	126	108	136	119	98	228	142	−0.297
Dec_‐1_	134	144	110	157	133	108	97	91	85	96	0.178
Jan	90	124	180	116	124	162	127	145	107	102	0.346
Feb	109	116	134	151	210	102	152	177	133	94	−0.256
Mar	193	193	160	181	130	111	176	231	232	156	−0.562
Apr	122	168	153	278	125	221	218	239	162	175	−0.531
Nov_‐1_–Apr	792	835	866	1009	830	840	889	981	947	765	−0.740*
Precipitation (mm)											
Sep_‐1_–Dec_‐1_	155	159	180	144	244	222	232	187	290	250	−0.113
Jan–Apr	143	133	160	98	212	161	93	62	212	175	0.293
FT (d)	49	58	68	47	69	63	69	57	46	36	0.417
Snow cover (d)	138	148	180	44	189	169	147	137	138	151	0.727*

Maximum loss of hydraulic conductivity (LC_max_; mean ± SE at the sampling date with highest values) and mean percentage loss of hydraulic conductivity (LC_J–A_; mean ± SE between January and April) and minimum water potential in winter (Ψ_min_; see Fig. 1 for *n*) as well as the following climate parameters are given (subscript ‐1 indicates months of the previous year, respectively): mean air temperature and sunshine duration in winter months of the previous (Nov_‐1_, Dec_‐1_) and respective year (Jan to Apr) as well as cumulative sunshine duration (Nov_‐1_–Apr), cumulative precipitation from September to December of the previous (Sep_‐1_–Dec_‐1_) and from January to April of the respective year (Jan–Apr), estimated number of days with freeze–thaw cycles (FT) and permanent snow cover during winter months (years refer to January, respectively). The number of days with freeze–thaw cycles was estimated by counting days with a daily minimum temperature lower than −3°C and daily sunshine duration higher than 50% of maximum day length. The snow cover duration gives the number of consecutive days with a minimum snow height of 10 cm. Data were correlated with LC_max_. Correlation was tested with Pearson´s correlation coefficient after testing for normal distribution (* indicates significant correlation). In case of nonnormal distribution (Sunshine Nov_‐1_, snow cover), correlation was tested with Spearman´s correlation coefficient. Climate data are from stations near the study site (Mount Patscherkofel 2252 m provided by ZAMG Zentralanstalt für Meteorologie und Geodynamik, Austria; Lämmerbichlalm 2020 m provided by Lawinenwarndienst Tirol, Austria).

In agreement with previous studies (Mayr *et al.*, 2003b, 2006a), the extent of winter embolism corresponded to minimum Ψ during winter months, which reached values down to −4.3 MPa (winter 2005; Fig. 1). Maximum LC and minimum Ψ were correlated when the winter of 2007 was excluded from analysis (*r* = −0.858, *n* = 9). Moreover, maximum LC, which was always reached between end of January and beginning of April, was negatively correlated with winter sunshine duration, with air temperatures in early (November) and late winter (March), and positively correlated with duration of the snow cover (including 2007; Table 1; Fig. S1). All these environmental factors enhance frost drought, as limited sunny days, low air temperatures in early and late winter and the resulting long‐lasting snow cover extend the period of blocked water uptake (due to frozen soil and/or stems). Though, even lowest Ψ observed cannot explain measured winter LC: based on the vulnerability curve for drought induced embolism, a Ψ of −4.3 MPa, as measured in winter 2005, corresponds to 41.6% LC, but led to 100% LC at the timberline (compare Fig. 1 and Fig. S1). On average, maximum field LC was 54.6 ± 7.8% higher than predicted from drought‐induced vulnerability over studied winter seasons. According to previous studies (Sparks & Black, 2000; Mayr *et al.*, 2003a,b) it is likely that freeze–thaw stress is a main cause for this increase in LC. Mayr *et al.* (2003a) demonstrated in young, potted *Picea abies* trees, that 100 freeze–thaw cycles can cause a 1.8 MPa increase in vulnerability thresholds and LC can thus be up to 100% higher under combined freeze–thaw/drought stress than under drought stress only. This combined stress action is also indicated in our data set as LC tended to increase with the number of freeze–thaw events (Table 1). The relationship was relatively weak, probably because the number of freeze–thaw events on the xylem could only be estimated from climate data. Overall, long‐term data underline that the combination of frost drought and freeze–thaw events caused high LC, whereby seasonal changes in xylem sap surface tension may further increase the risk of embolism formation in winter (Losso *et al.*, 2017). According to experimental studies (Brodribb & Cochard, 2009; Brodribb *et al.*, 2010; Choat *et al.*, 2018) more than 50% LC are critical for conifers, while a recent study on *Pinus taeda* reported a mortality threshold of 80% LC (Hammond *et al.*, 2019). It is unclear if this experimental evidence (summer drought simulated on young, potted trees) can reflect winter situation at the timberline, and it is likely that mortality thresholds are species‐specific. Though, these findings clearly indicate that observed LC of up to 100% cause a dramatic situation for timberline conifers; unless they are able to repair their hydraulic system before the vegetation period starts.

### Repair of winter embolism

Our long‐term observations demonstrate that timberline conifers are definitely able to recover from winter embolism, which is obvious from Fig. 1: LC during summer was always lower than in the previous winter season, indicating annual recovery from winter embolism. In fact, maximum LC during summer never exceeded 16%, which proves an efficient recovery from winter embolism as suggested in some previous studies. A stepwise, several weeks lasting recovery has been reported for several conifer genera such as *Abies*, *Juniperus*, *Larix*, *Pinus*, *Pseudosuga*, *Thuja* and *Tsuga* (Sperry *et al.*, 1994; Sparks & Black, 2000; Mayr *et al.*, 2006a; McCulloh *et al.*, 2011). In timberline trees of the Central European Alps, studies indicated recovery to start in late winter and to continue until early summer (Mayr *et al.*, 2003b, 2006a, 2014). Mayr *et al.* (2014) found that refilling was supported by branch water uptake, probably from melting snow as also suggested for *Pinus contorta* during recovery from winter embolism (Sparks *et al.*, 2001). Foliar water uptake was also reported for species of *Sequoia sempervirens* forests, which can absorb water from fog (Burgess & Dawson, 2004; Limm *et al.*, 2009), and experimentally demonstrated on *Pinus glauca* (Laur & Hacke, 2014). Earles *et al.* (2016) reported water uptake via the bark. In timberline conifers, Mayr *et al.* (2014) suggested active cellular processes, including aquaporin activity (Sakr *et al.*, 2003; Secchi & Zwieniecki, 2010, 2012) to facilitate the refilling process. As reviewed in Berry *et al.* (2019), Ψ can rapidly increase upon foliar water uptake, with changes up to *c*. 0.1 MPa min^–1^, as observed in *Juniperus monosperma* (Breshears *et al.*, 2008).

**Figure 2:**
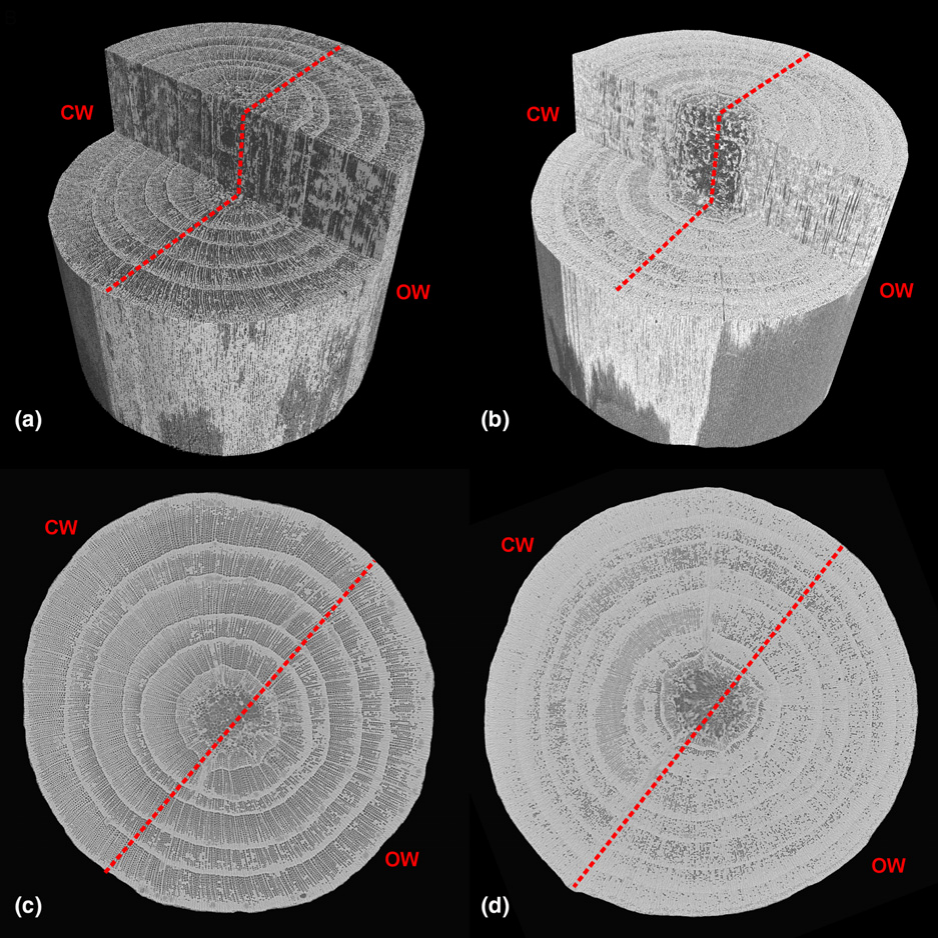
X‐ray microtomographic observation of branch stems harvested in April (a, c), and August 2013 (b, d)*.* Stems were oriented with the compression wood (CW) in the background and opposite wood (OW) in front, respectively. Black lumina in the xylem indicate air‐filled, white areas water‐filled conduits. Upper panels show three‐dimensional reconstructions and lower panels respective two‐dimensional cross‐sections.

Our direct micro‐CT observations confirm that recovery from winter‐embolism was based on xylem refilling. Fig. 2 shows an exemplary 3D‐reconstruction of branch samples harvested in late winter and summer 2013 (note that respective tree rings thus reflect the ‘hydraulic history' according to presented long‐term data on LC and Ψ). Micro‐CT images of branches in winter revealed a uniformly embolized xylem with only a few groups of functional tracheids in the first conduit rows (earlywood) of each tree ring. In conduits formed later in the vegetation period (latewood), most of the tracheids were water‐filled and, in consequence, responsible for part of the remaining conductivity. Remarkably, this pattern was identical in every tree ring, and quantification of conductivities from filled and embolized tracheids revealed only a small variation in LC across tree rings. The mean calculated LC averaged for each tree ring was 72.7 ± 2.3%. This indicates similar stress intensity but also similar vulnerability in the entire cross‐section of branches and thus no weakening of the xylem caused by previous embolism events. Such an increase in vulnerability against drought stress after embolism formation and repair (cavitation fatigue) was first described by Hacke *et al.* (2001) for *Acer*, *Alnus* and *Populus* species and may also occur after freeze–thaw induced embolism (Feng *et al.*, 2015) and in conifers (Torres‐Ruiz *et al.*, 2016). Thus, the homogenous LC in sample cross‐sections (Fig. 2a) remains surprising, considering that older tree rings were exposed to overall more embolism‐refilling cycles than younger ones (Fig. 1) and, in case of cavitation fatigue, LC should have increased towards the centre of the branches. The lack of such a trend in LC suggests that xylem vulnerability was not influenced by the preceding number of embolism and refilling cycles. This was further supported by complementary laboratory experiments on Norway spruce branches harvested at the timberline (Fig. S2): in stem samples, 50% and 100% LC were induced before they were rehydrated via vacuum infiltration. Vulnerability after this embolism‐refilling cycle was identical to the vulnerability of controls. It remains to be studied if cavitation fatigue in conifers is species‐ and/or site‐specific and how the fragile pit architecture of timberline conifers can withstand such extraordinary and repeated stresses.

In summer, micro‐CT images of branches showed overall low amounts of embolized tracheids (Fig. 2b). Interestingly, and in contrast to patterns in winter, the water content in cross‐sections was not homogenous. While the outermost tree ring was nearly completely water filled (9% and 20% calculated LC in the two samples), inner rings contained distinct areas with air filled tracheids (mean calculated LC across inner tree rings 36.0 ± 5.8% and 38.5 ± 3.1%), especially in the compression wood (Mayr & Cochard, 2003). No trend between these inner tree rings was observed indicating that hydraulic constraints developed between the first and second year but not in consecutive years. It suggests that newly formed xylem contains a portion of comparably vulnerable tracheids, which lose their hydraulic functionality during the first winter and which cannot be refilled. Though, it is remarkable that a major fraction of conductivity in older tree rings was maintained throughout years despite repeated winter embolism.

## Conclusion and remaining knowledge gaps

Our long‐term data provide important insights regarding underlying processes and the relevance of xylem embolism as well as counter strategies of plants. Intra‐ and inter‐rings water status analyses also show that refilling, besides embolism avoidance, has to be taken into account as a key strategy of timberline trees to overcome hydraulic limitations caused by the combination of frost drought and freeze–thaw stress during winter. Timberline trees thereby are an example for very extreme conditions and effects on xylem hydraulics, but the analyses of these eco‐physiological limits may help to better understand general plant hydraulics. In each case, more field studies, more long‐term studies and more studies on adult trees are required to enable an interpretation of short‐term, laboratory and experimental findings and comprehensively understand the ecology of tree hydraulics.

Based on the presented study, several knowledge gaps with respect to winter embolism can be identified: first, the process of ice formation in plant xylem and how it leads to embolism is still not sufficiently understood (e.g. Lintunen *et al.*, 2014, 2018; Charrier *et al.*, 2017). Considering the relevance of high altitude and latitude forest ecosystems, substantial knowledge of the eco‐physiological limits of trees is important, and information on freezing‐related mechanisms a prerequisite. Second, the effects of combined stresses on xylem hydraulics are not well known: this is true for the combination of freeze–thaw events and drought analysed in the present study, but also, for instance, on the combination of freezing and mechanical stress (e.g. Christensen‐Dalsgaard & Tyree, 2013), drought and mechanical stress (Niez *et al.*, 2019) or drought and pathogen stress (e.g. Anderegg *et al.*, 2015a; Reblin & Logan, 2015). Combined stresses occur frequently *in natura*, and resulting effects may substantially differ from impairments caused by single stress factors. Third, there is only a limited number of hydraulic studies on repeated stresses and hydraulic resilience and legacy effects (e.g. Anderegg *et al.*, 2015b; Schwalm *et al*., 2017; Yin & Bauerle, 2017). For instance, only scarce information regarding cavitation fatigue, as analysed in our study, is available (Hacke *et al.*, 2001; Feng *et al.*, 2015; Hillabrand *et al.*, 2016; Torres‐Ruiz *et al.* 2016). A recent study (Umebayashi *et al.*, 2019) indicated that fluctuation of sap Ψ, even above the critical thresholds for embolism formation, might weaken the xylem. These aspects are especially relevant in the light of climate change, as the future distribution of trees species (e.g. Allen *et al.*, 2010; Zwieniecki & Secchi, 2015; Choat *et al.*, 2018; Jump *et al.*, 2017) will depend on their reactions to consecutive stress events. Timberline trees might profit from higher mean temperatures, which will decrease the duration of winter droughts, while changes in precipitation may lead to reduced snow cover protection and an increase in temperature extremes to even higher numbers of freeze–thaw cycles. Fourth, the role of hydraulic architecture and hydraulic segmentation (e.g. Tyree & Zimmermann, 2002; Hochberg *et al.*, 2016) during and after hydraulic stress is still not well studied. The majority of studies (for methodical reasons) focused on branch xylem (also see McCulloh *et al.*, 2019), while information on other parts of the axes system and especially the main stem of trees remains rare, despite its relevance in the hydraulic pathway or capacitance effects, which are crucial during winter. We also encourage studies on smaller scales, such as comparing the hydraulic safety and efficiency between branch orders, junctions and intersections, between and within tree rings. Respective studies will shed light on the limits of tree hydraulics under present and future climatic conditions, and show if trees will still die hard.

## Author contributions

SM was the leading investigator in this project. SM and PS performed fieldwork and hydraulic measurements for seasonal courses. FF was responsible for cavitation fatigue analysis and EB for X‐ray micro‐CT visualization. BB helped with data analysis. SM, BB and EB prepared the manuscript.

## Supporting information


**Fig. S1** Correlation of maximum loss of conductivity with hydraulic and climate parameters according to Table 1.
**Fig. S2** Test of cavitation fatigue in stem samples after induction of 50% or 100% loss of conductivity and refilling.
Please note: Wiley Blackwell are not responsible for the content or functionality of any Supporting Information supplied by the authors. Any queries (other than missing material) should be directed to the *New Phytologist* Central Office.
