# Supplementary material for: Die hard: timberline conifers survive annual winter embolism
Source: New Phytol. 2019 Nov 23;226(1):13–20. doi: 10.1111/nph.16304 (PMC7065000; doi:10.1111/nph.16304)
Supplement: Supplementary file 1 — Fig. S1 Correlation of maximum loss of conductivity with hydraulic and climate parameters according to Table 1. Fig. S2 Test of cavitation fatigue in stem samples after induction of 50% or 100% loss of conductivity and refilling. Please note: Wiley Blackwell are not responsible for the content or functionality of any Supporting Information supplied by the authors. Any queries (other than missing material) should be directed to the New Phytologist Central Office. [file NPH-226-13-s001.pdf]

New Phytologist Supporting Information

# Die hard: timberline conifers survive annual winter embolism

Stefan Mayr, Peter Schmid, Barbara Beikircher, Feng Feng, Eric Badel

Article acceptance: 27 October 2019

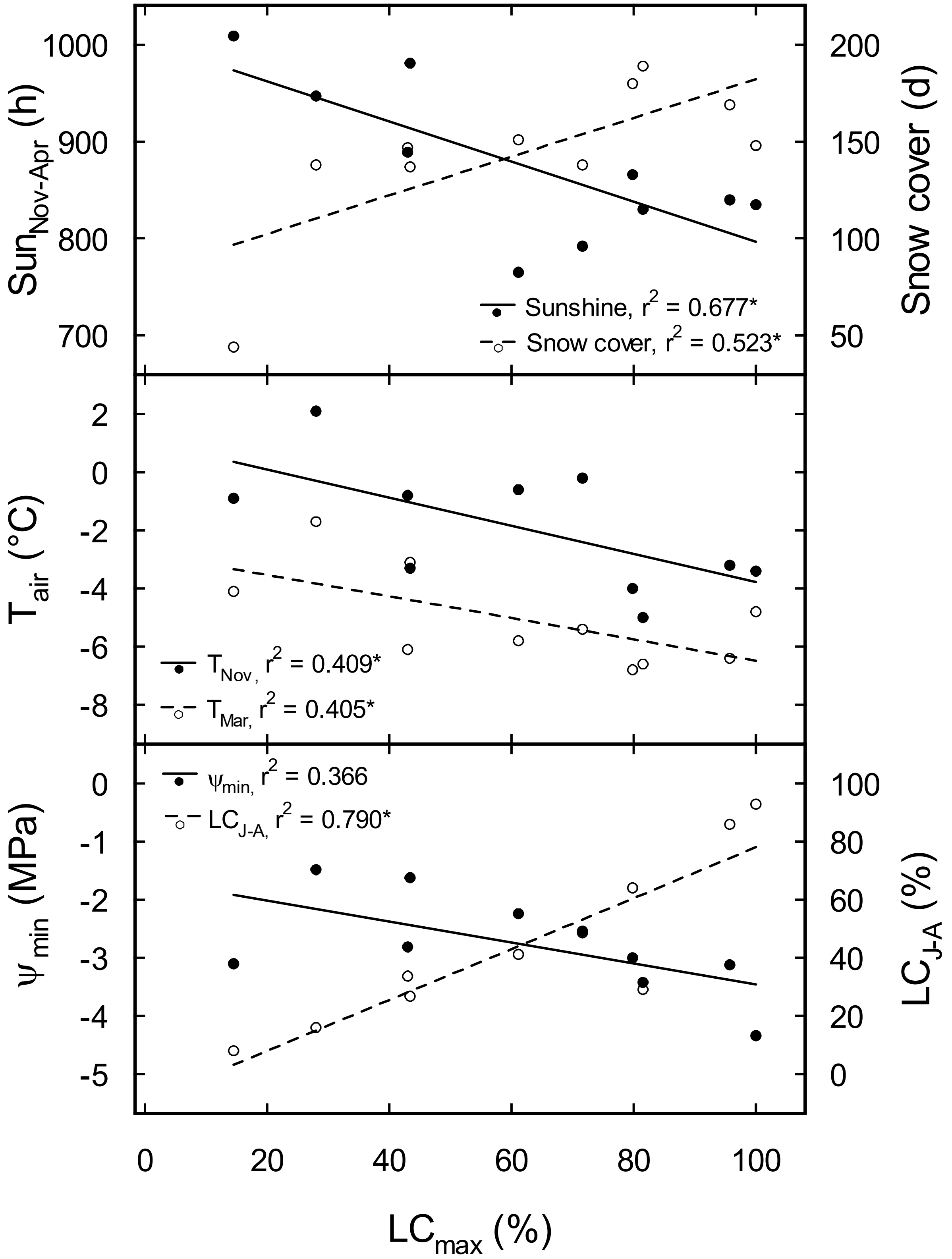


**Figure S1: Correlation of maximum loss of conductivity with hydraulic and climate parameters.** Mean maximum percent loss of conductivity ($LC_{max}$) was correlated with the cumulative sunshine duration from November to April ($Sun_{Nov-Apr}$) and duration of snow cover, air temperature in November and March ($T_{Nov}$, $T_{Mar}$) and with minimum water potentials ($\Psi_{min}$) and mean loss of conductivity between January and April ($LC_{J-A}$). Data according to Table 1.

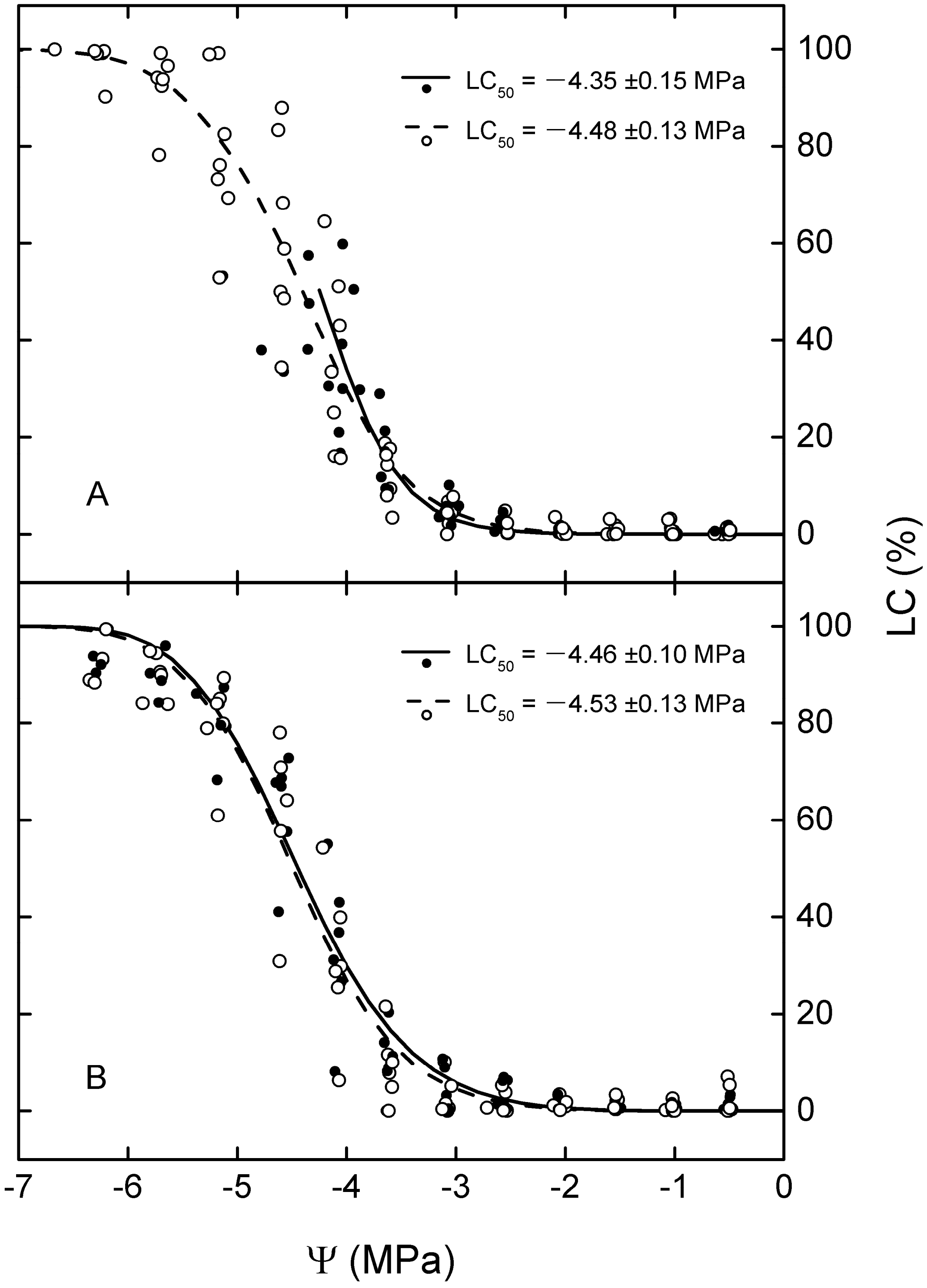


**Figure S2: Test of cavitation fatigue.** Branch stems were exposed to decreasing water potential (Ψ) until 50 and 100% loss of conductivity (LC) were reached (solid lines and dots in panel A and B, n= 7 and 6, respectively; note that the vulnerability curve in panel A stops when 50% LC was reached). After rehydration *via* vacuum infiltration, a second (entire) vulnerability curve (dashed lines and open dots) of identical samples was measured. Mean exponential curve fittings are given. $LC_{50}$ is the average Ψ at 50% LC calculated from individual vulnerability curves.
